# Recognition mechanisms of hemoglobin particles by monocytes – CD163 may just be one

**DOI:** 10.3762/bjnano.14.85

**Published:** 2023-10-19

**Authors:** Jonathan-Gabriel Nimz, Pichayut Rerkshanandana, Chiraphat Kloypan, Ulrich Kalus, Saranya Chaiwaree, Axel Pruß, Radostina Georgieva, Yu Xiong, Hans Bäumler

**Affiliations:** 1 Institute of Transfusion Medicine, Charité-Universitätsmedizin Berlin, Berlin, Germany; 2 Division of Clinical Immunology and Transfusion Sciences, School of Allied Health Sciences, University of Phayao, Phayao 56000, Thailand; 3 Department of Pharmaceutical Technology and Biotechnology, Faculty of Pharmacy, Payap University, Chiang Mai, Thailand; 4 Department of Medical Physics, Biophysics and Radiology, Medical Faculty, Trakia University, Stara Zagora 6000, Bulgaria

**Keywords:** CD163, HBOC, hemoglobin-based oxygen carriers, monocytes, phagocytosis

## Abstract

Hemoglobin-based oxygen carriers (HBOCs) as blood substitutes are one of the great hopes of modern transfusion and emergency medicine. After the major safety-relevant challenges of the last decades seem to be largely overcome, current developments have in common that they are affected by degradation and excretion at an early stage in test organisms. Several possible mechanisms that may be responsible for this are discussed in the literature. One of them is CD163, the receptor of the complex of haptoglobin (Hp) and hemoglobin (Hb). The receptor has been shown in various studies to have a direct affinity for Hb in the absence of Hp. Thus, it seems reasonable that CD163 could possibly also bind Hb within HBOCs and cause phagocytosis of the particles. In this work we investigated the role of CD163 in the uptake of our hemoglobin sub-micron particles (HbMPs) in monocytes and additionally screened for alternative ways of particle recognition by monocytes. In our experiments, blockade of CD163 by specific monoclonal antibodies proved to partly inhibit HbMP uptake by monocytes. It appears, however, that several other phagocytosis pathways for HbMPs might exist, independent of CD163 and also Hb.

## Introduction

Blood transfusions save lives every day and have become an indispensable part of clinical practice in modern medicine. However, there have always been numerous limitations and problems with their use, namely dependence on donor readiness, short shelf life [1], risk of infection [2], transfusion reactions in the case of non-blood group-specific transfusion [3], immunization with the formation of antibodies [4], and an overall high personnel and financial expense [5].

For decades, science has been trying to overcome these problems [6]. Although erythrocytes also pose as a CO_2_ transporter and native hemoglobin as a buffer system [7], research is being focused on their function as oxygen transporters. The search is on for a laboratory-produced oxygen carrier that is relatively simple and inexpensive to produce, can be stored for a long time and administered universally, is safe to use, and has few to none adverse drug effects.

One approach associated with great hopes are hemoglobin-based oxygen carriers (HBOCs). Initially, however, serious side effects were encountered during development, namely nitrogen monoxide scavenging and associated hypertensive crises, massive renal damage due to tubular reabsorption of hemoglobin (Hb), decay into dimers [8–13], and oxidative stress [14–15]. Various approaches of intra- as well as intermolecular modifications of HBOCs have been devised. Crosslinking of different Hb chains, polymerization of Hb molecules [16–18], surface modification [19], and techniques for encapsulation [20–21] already brought us somewhat closer towards a safe use of HBOCs [22]. However, a persistent problem is the severely limited retention time of oxygen carriers in the circulatory system. Erythrocytes and the Hb they contain have a mean survival time of about 120 days within the human body [7]. HBOCs, in contrast, have been eliminated within minutes to a few hours in previous experiments [5,23]. Much of this process appears to occur in the liver [24–26]. The question of the mechanisms by which HBOCs are sequestered remains partly unclear though.

Possible degradation pathways include haptoglobin (Hp), which, depending on the size and surface properties of HBOCs, could bind its physiological target protein hemoglobin [16,19]. CD163, the receptor for Hp–Hb complexes also shows some affinity for Hb [16,27–28]. The corresponding binding site at the receptor appears to be the same as for the binding of Hp–Hb complexes, according to Schaer et al. [27]; whereas within Hb, the binding site for direct interaction with CD163 is probably within the β chain of Hb (binding of Hb to Hp via a binding site within the Hb α chain) [27]. Furthermore, not only cells of the monocyte/macrophage lineage appear to be involved in sequestering Hb, but also hepatocytes [25–26]. It can be speculated that the same could be true for HBOCs. This fits with a case report by Drieghe et al., which suggests that hemopexin (Hpx) may also play a role in the elimination of HBOCs [25,29]. Hpx was depleted before a change in Hp levels could be observed when catecholamine-requiring patients were treated with an HBOC for intentional NO scavenging and consecutive increase in peripheral vascular resistance. Whether binding between cell surface proteins and Hb/HBOC can occur, and how high the corresponding affinity is, probably depends on modifications made to Hb. Intramolecular crosslinking has an impact, depending on whether the binding site within the Hb α chain is freely accessible (exclusively β-crosslinked Hb vs α-crosslinked Hb) [27]. Intermolecular modifications changing the molecular or polymer size of HBOCs [16,19,30] are relevant for protein binding as well. Hemoglobin sub-micron particles (HbMPs) obtained via coprecipitation–crosslinking–dissolution (CCD) are promising as HBOCs. CCD provides particles that are malleable and show a consistent morphology and narrow size distribution, as well as a negative zeta potential [19,31–33]. It could be shown that neither NO scavenging nor vasoconstriction can be detected when using HbMPs as oxygen carriers [34]. In addition to transporting oxygen, HbMPs can also be used as drug carriers. However, in a pharmacokinetic study with HbMPs, accumulation of the particles in the sinusoids of the liver, where the Kupffer cells are located, was observed [35]. The mechanisms of the interaction of liver macrophages with HbMPs have not been systematically investigated yet. Since HbMPs are composed of Hb, the elimination via Hp and Hpx seems likely. Hp binds freed Hb, Hpx binds freed heme. The resulting complexes are then bound by the respective receptors, namely CD163 for Hp and CD91 for Hpx, and taken up by phagocytes (e.g., Kupffer cells in liver sinoids), where Hb or heme are subsequently degraded. If this mechanism cannot be bypassed, Hp and Hpx must be fully saturated to achieve and maintain the effect of the HBOCs.

Here is an example calculation with commercially available HBOC 201 (Hemopure^®^; hemoglobin glutamer-250 (bovine); Hemopure, HbO2 Therapeutics LLC, Souderton PA 18964, USA): A dose of 60 g HBOC 201 in 5 L of blood results in an HBOC concentration of 2.9 × 10^19^ HBOCs/mL. Hp serum concentrations amount in average to about 1.5 g/L; this corresponds to 1 × 10^15^ molecules/mL. The average Hpx serum concentration of 0.6 g/L corresponds to 4.5 × 10^15^ molecules/mL. Thus, the dose of 60 g HBOC 201 is sufficient to bind the available Hp and Hpx, allowing subsequent doses of HBOCs to remain in circulation until these proteins are replenished. In case of infusion with HbMPs of 20% (v/v) in reference to a blood volume of 5 L, this would correspond to a concentration of 2.3 × 10^11^ HbMPs/mL. In this case, Hp and Hpx would still be present in excess and could thus bind and eliminate all HbMPs. The need arises to verify whether it is indeed Hp and/or Hpx that are responsible for the elimination of HbMPs. A direct interaction with the monocyte receptor CD163, as already described in the literature for other Hb derivatives, is also conceivable. Another possibility would be an elimination of HbMPs independent of the Hb content, which is instead influenced by particle size or other physical properties of the particles.

In this study, we screened several monocytic surface receptors for a possible influence on the uptake of HbMPs by monocytes, which are precursor cells of macrophages. We chose to screen for CD14- as well as CD33-dependent HbMP uptake by monocytes since both proteins are specific for monocytes. CD163 was tested because of its direct affinity to Hb. Also, we tested the effect of blocking CD204 (scavenger receptor A/SR-A). SR-A is a membrane protein occurring in the monocyte/macrophage lineage. Playing an important role in host defense, it exhibits a long list of ligands including modified serum albumin, which might also occur within our HBMPs [36]. The interaction of HbMPs (with different surface modifications) with Hp, as well as with anti-Hb antibodies has already been studied by Prapan and co-workers [19]. With this work, the investigation is extended to the role of CD14, CD33, CD163, and CD204 in the uptake of HbMPs by monocytes. We decided to not research the effect of Hpx on HbMPs in this study, since there is no indication for heme to be accessible within our HbMPs. We hypothesize that CD163 plays an important role in the uptake of our HbMPs into monocytes, while there might be additional other mechanisms at play. The aim of this work is to contribute a further step towards the development of HbMPs as a complete, safe blood substitute for volume expansion and oxygen distribution.

## Materials and Methods

### Chemicals

0.9% sodium chloride was purchased from B. Braun SE, Melsungen, Germany. Phosphate-buffered saline (PBS) 10-fold solution was purchased from Fisher scientific, Fair Lawn, New Jersey, USA. Human serum albumin (Plasbumin^®^20) was purchased from Grifols Deutschland GmbH, Frankfurt am Main, Germany. Trypan blue 0.4% solution was purchased from PAN-Biotech GmbH, Aidenbach, Germany. Manganese chloride, sodium hydroxide, sodium borohydride, sodium carbonate, ethylenediaminetetraacetic acid (EDTA), glutaraldehyde solution, grade II, 25% in H_2_O, as well as Triton™ X-100 were purchased from Sigma-Aldrich, Steinheim, Germany. Pronase was purchased from Roche diagnostics GmbH, Mannheim, Germany. Bovine hemoglobin (Actoheme^®^) was provided by Biophyll GmbH, Dietersburg, Germany.

PHAGOTEST™ and PHAGOBURST™ test kits were purchased from Glycotope Biotechnology GmbH, Berlin, Germany. Mouse-derived monoclonal anti-human CD14 antibody (IgG2a, Κ), conjugated with fluorescein isothiocyanate (FITC) was purchased from BD Biosciences, Heidelberg, Germany. Mouse-derived monoclonal anti-human CD14 antibody (IgG2a, Κ) was purchased from BioLegend, San Diego, USA. Mouse-derived monoclonal anti-human CD33 antibody (IgG1, K) was purchased from BioLegend, San Diego, USA. Mouse-derived monoclonal anti-human CD163-antibody (IgG1, clone: GHI61), conjugated with allophycocyanin (APC) was purchased from antibodies-online GmbH, Aachen, Germany. Mouse-derived anti-human CD204-antibody (IgG2a, K) was purchased from BioLegend, San Diego, USA.

### Donated blood

Peripheral venous blood was collected from healthy donors and anticoagulated with lithium heparin according to the requirements of the German law regulating transfusion. Written informed consent was obtained from all donors, as well as a positive vote of the Ethics Committee of the Charité - Universitätsmedizin Berlin (EA4/023/22). Blood samples were processed immediately after blood collection, and cell metabolism was maximally slowed down by storage at 0 °C.

### Particle preparation

For the preparation of hemoglobin-based sub-micron particles (HbMPs), the protocol of particle preparation using the CCD technique previously described [24] has been followed: 0.5% bovine hemoglobin solution was mixed with 0.125 M MnCl_2_ solution. While stirring rapidly, 0.125 M Na_2_CO_3_ was added and stirred further for 30 s to let Hb–MnCO_3_ particles form. 20% human serum albumin (HSA) solution was added and allowed to incubate with the particles for 5 min. After washing in 0.9% NaCl solution, particles were centrifuged (3000*g*, 3 min), and the supernatant was decanted. Particles were then resuspended in 0.9% NaCl. For crosslinking, 0.02% glutaraldehyde (GA) was added to the particle suspension, which was then incubated for 1 h at room temperature under stirring. After another washing in aqua dest. the MnCO_3_ matrix was dissolved using 0.25 M EDTA. After 30 min incubation time, 0.2 mg/mL NaBH_4_ in 0.1 M NaOH was added to prevent oxidation of Hb to met-Hb. Incubation for another 30 min followed. After triplicate washing in a washing solution of 0.9% NaCl containing 0.2% HSA, particles were resuspended in 0.9% NaCl, checked via light microscopy for aggregation, and stored at 4 °C.

### Particle characterization

To ensure that the subsequent experiments would be carried out on particles that also met the quality requirements for potential use as oxygen transporters in vivo, the particles were characterized in different ways. Thus, comparability was achieved between different particle batches in different experiments. All experiments within this work have been performed with HbMPs from the same batch.

### Concentration determination

HbMP suspension was placed in glass capillaries for hematocrit determination and then centrifuged at 15,000*g* for 10 min. The amount of particle sediment was determined manually, and the remaining suspension was diluted with 0.9% NaCl to a concentration of 2% HbMP.

### Size, zeta potential, and conductivity

After dilution of the particle suspension to 0.13% (V/V) with NaCl, the average size and conductivity, as well as the zeta potential of the particles were determined using a zetasizer (zetasizer Nano ZS, Malvern Instruments, Malvern, United Kingdom) with each measurement in triplicate.

### Hemoglobin content

To determine the HbMPs’ hemoglobin content, the modified alkaline hematin-D method (AHD method) was used, as described in detail elsewhere [33,37]. Pronase solution (0.5 mg/mL) was added to the particle suspension and incubated at 45 °C for 30 min. The AHD reagent (25 mg/mL Triton™ X-100 in 0.1 M NaOH) was added to the particle sample (final concentration: 12.5 mg/mL), mixed, and incubated at room temperature for 15 min while stirring. After centrifugation (20,000*g*; 10 min) the supernatant was taken off and, immediately, the absorbance at a wavelength of 574 nm was determined photometrically in three single measurements (cytation 3 imaging reader, BioTek Instruments GmbH, Bad Friedrichshall, Germany). The hemoglobin concentration of the particles was then calculated from the measured values according to the following formula, as published by Smuda et al. [38]:


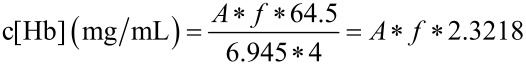


(*A* = absorbance at 574 nm; ƒ = dilution factor (2.1); molar mass of Hb (tetrameric) = 64.5; (monomer) molar extinction coefficient (ε) = 6.945).

### Percentage of functional hemoglobin

Each hemoglobin molecule contains an Fe^2+^ ion on which the ability of Hb to bind oxygen is based. Methemoglobin has undergone an oxidation process. The central iron ion is trivalent, and the Hb derivative has lost its ability to bind oxygen. Therefore, only bivalent Hb is functional. The “oxygen release method” was used to determine the percentage of functional hemoglobin, as previously described in detail by Kloypan and co-workers [33]. In brief, the oxygen content of the suspension was measured in three individual measurements in 500 µL each of the particle suspension diluted to a total Hb content of 0.5 mg/mL under steady, gentle stirring (Microx 4, PreSens, Regensburg, Germany). After reaching a stable initial value, 50 µL of 10% potassium ferricyanide (K_3_[Fe(CN)_6_]) was added, and the stirring speed was increased. The oxygen bound to Hb was thus released, and the peak value of O_2_ within the suspension was determined. From the difference between initial and peak values, the proportion of functional Hb was calculated.

### Indirect phagocytosis test

In the development of a suitable experimental setup, a strong influence of direct fluorescent labeling of HbMPs on the phagocytosis activity of monocytes was found. Consequently, we chose an indirect phagocytosis assay for our experiments, which did not require any labeling of the HbMPs potentially to be phagocytosed. The method of the indirect phagocytosis assay is described in detail elsewhere [22] and is illustrated in Supporting Information File 1, Figure S1.

In brief, in indirect phagocytosis test no. 1, samples were prepared each with and without an insert of 5 µL of anti-CD163-AB (APC-conjugated) to block the monocytic membrane protein, as formerly described by Schaer and co-workers [27]. For a reference sample, the phagocytic capacity of monocytes was fully utilized for the uptake of FITC-labeled *E. coli* lysate over a period of 10 min at 37 °C (commercially available phagocytosis tests use lysate rather than whole bacteria for the higher phagocytosis efficacy of lysates). For comparison, samples were prepared in which the cells were able to phagocytose either unlabeled *E. coli* lysate or HbMP in a pre-feeding step (approximately 100 HbMPs per leukocyte). The incubation period here was 120 min. In addition, samples with HbMP pre-feeding were also prepared with a shortened incubation time of only 30 min to obtain information of the course of phagocytic activity over time. Table 1 offers an overview of the samples described above. Identical negative controls were carried along, which remained at 0 °C at all times (Supporting Information File 1, Figure S1).

**Table 1:** Samples for indirect phagocytosis test no. 1, role of CD163.

Sample^a^	AB use	pre-fed with	Purpose

reference	no	no	max. MFI
EC-prefed-120′	no	*E. coli*, untagged	Extent of *E. coli*-phagocytosis
CD163-block	yes	no	AB-impact on FITC-*E. coli* phagocytosis
CD163-block+EC-prefed-120′	yes	*E. coli*, untagged	AB-impact on untagged *E. coli* phagocytosis
HbMP-prefed-30′	no	HbMP	Unhindered uptake of HbMP, 30 min
HbMP-prefed-120′	no	HbMP	Unhindered uptake of HbMP, 120 min
CD163-block+HbMP-prefed-30′	yes	HbMP	uptake of HbMP with blocked CD163, 30 min
CD163-block+HbMP-prefed-120′	yes	HbMP	uptake of HbMP with blocked CD163, 120 min

^a^In a second step, all indirect phagotest samples were also incubated with FITC-tagged *E. coli* for 10 min at 37 °C; EC = *E. coli*; AB = antibody.

Analogous to CD163, samples were prepared in which CD14, CD33, CD204, or all of these monocytic surface proteins were blocked using specific antibodies (indirect phagocytosis test no. 2). These samples allowed for an assessment of the dependence of HbMP uptake on these receptors. Table 2 gives an overview of the samples of indirect phagocytosis test no. 2; the incubation time with HbMPs was 30 min. All samples were analyzed by flow cytometry. Leucocytes were identified by DNA staining with propidium iodide (PI) or diamidinophenylindole (DAPI). The closely spaced emission maxima of DAPI and FITC, or PI and APC, necessitated the use of different dyes for DNA staining depending on which signal, FITC or APC, was of interest in each sample. In each run, 2000 monocytes were analyzed. The mean fluorescence intensity (MFI) of the reference sample and the corresponding reduction in the other samples provided information about the extent of phagocytosis in the pre-feeding step. For each experimental run, blood from a different donor was used. Due to the donors’ variability (e.g., different cell counts, immune cell activity, or surface receptor density), a relatively wide spread of MFI values between the single runs was observed, while relations between the different samples and their respective reference sample within one run were rather stable. Data were therefore normalized by calculating each sample’s MFI, relative to its reference within one run (expressed as percentages). Then the arithmetic means over three experimental runs were calculated from these normalized MFI values.

**Table 2:** Samples for indirect phagocytosis test no. 2, role of CD14, CD33, and CD204.

Sample^a^	AB use	pre-fed with	Purpose

reference	no	no	max. MFI
HbMP-prefed-30′	no	HbMP	unhindered uptake of HbMP, 30 min
CD14-block	yes	no	AB-impact on FITC-*E. coli* phagocytosis
CD33-block	yes	no	AB-impact on FITC-*E. coli* phagocytosis
CD204-block	yes	no	AB-impact on FITC-*E. coli* phagocytosis
CD14,33,163,204-block	yes	no	AB-impact on FITC-*E. coli* phagocytosis
CD14-block+HbMP-prefed-30′	yes	HbMP	uptake of HbMP with blocked CD14, 30 min
CD33-block+HbMP-prefed-30′	yes	HbMP	uptake of HbMP with blocked CD33, 30 min
CD204-block+HbMP-prefed-30′	yes	HbMP	uptake of HbMP with blocked CD204, 30 min
CD14, 33, 163, 204-block+HbMP-prefed-30′	yes	HbMP	uptake of HbMP with blocked CD14, 33, 163, 204; 30 min

^a^In a second step, all indirect phagotest samples were also incubated with FITC-tagged *E. coli* for 10 min, at 37 °C; EC = *E. coli*; AB = antibody.

Furthermore, to identify the monocyte population in fluorescence-activated cell sorting (FACS) analysis, whole blood samples were each incubated with FITC-labeled mouse anti-human CD14 antibody (anti-CD14-wb) and APC-labeled mouse anti-human CD163 antibody (blocking antibody) (anti-CD163-wb); also, a whole blood sample lysed and DNA-stained only was prepared for comparison (wb). In addition, a whole blood sample that had been kept at 37 °C for 120 min, as the pre-fed samples were, was prepared to check for an influence of continued active cell metabolism on the MFI. Table 3 gives an overview of these samples.

**Table 3:** Samples for exclusion of confounders and identification of leucocyte subpopulations.

Sample^a^	Incubation with	Purpose

HbMPs	—	HbMP autofluorescence
DAPI-HbMP	DAPI	DAPI impact
PI-HbMP	PI	PI impact
wb	—	background noise
incub-wb	—	Impact of incubation time
DAPI-wb	DAPI	DAPI impact
PI-wb	PI	PI impact
anti-CD14-wb	Anti-CD14, FITC	identification of monocyte population
anti-CD163-wb	Anti-CD163, APC	identification of monocyte population

^a^wb = (human) whole blood.

## Results

### Particle characterization

A HbMP suspension was initially adjusted to a concentration of 2% (V/V) HbMPs as described. The hemoglobin content in this suspension was 5.4 ± 0.02 mg/mL. Analysis by zetasizer showed an average particle size of 781 ± 7 nm, a conductivity of 1.34 ± 0.07 mS/cm, and a zeta potential of −28.0 ± 0.5 mV. The oxygen release method measurement showed a percentage of 81.4% ± 2.13% functional Hb. Light microscopy showed no aggregation within the particle suspension.

### Exclusion of confounders, indirect phagocytosis test

**Figure 1:**
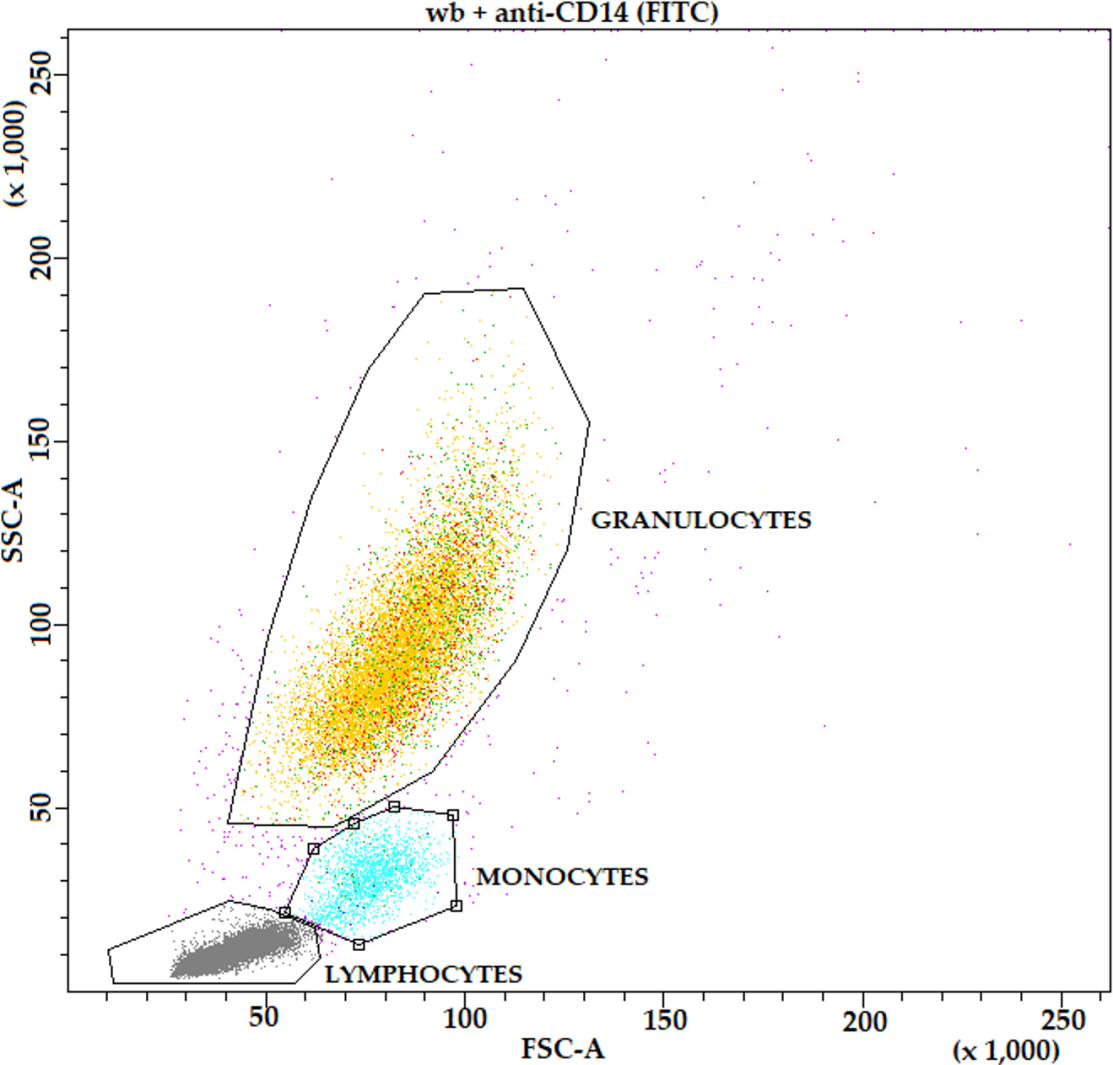
Leukocyte subpopulations, DNA-stained, FITC-tagged anti-CD14 AB (for better readability, all captions of the graphic have been enlarged. The dot plot itself has not been changed in any way).

HbMPs showed a low autofluorescence in the FITC channel in FACS analysis, which did not influence the fluorescence of DAPI in the PI channel. Whole blood (wb) generated a very low FITC signal as well. Neither holding a temperature of 37 °C for 120 min (Incub-wb) nor incubation with DAPI or PI (DAPI-/PI-wb) caused a relevant change in this signal. Neither the blood itself nor the HbMPs or the chosen incubation times had an interfering influence on the test. Staining the monocytes with FITC-labeled anti-CD14 antibody (anti-CD14-wb; 81.5 ± 2.3% FITC-positive monocytes) and APC-labeled anti-CD163 antibody (Anti-CD163-wb; 64.3 ± 3.8% APC-positive monocytes) revealed a clearly distinguishable monocyte population, as Figure 1 shows.

### Indirect phagocytosis test no. 1

**Figure 2:**
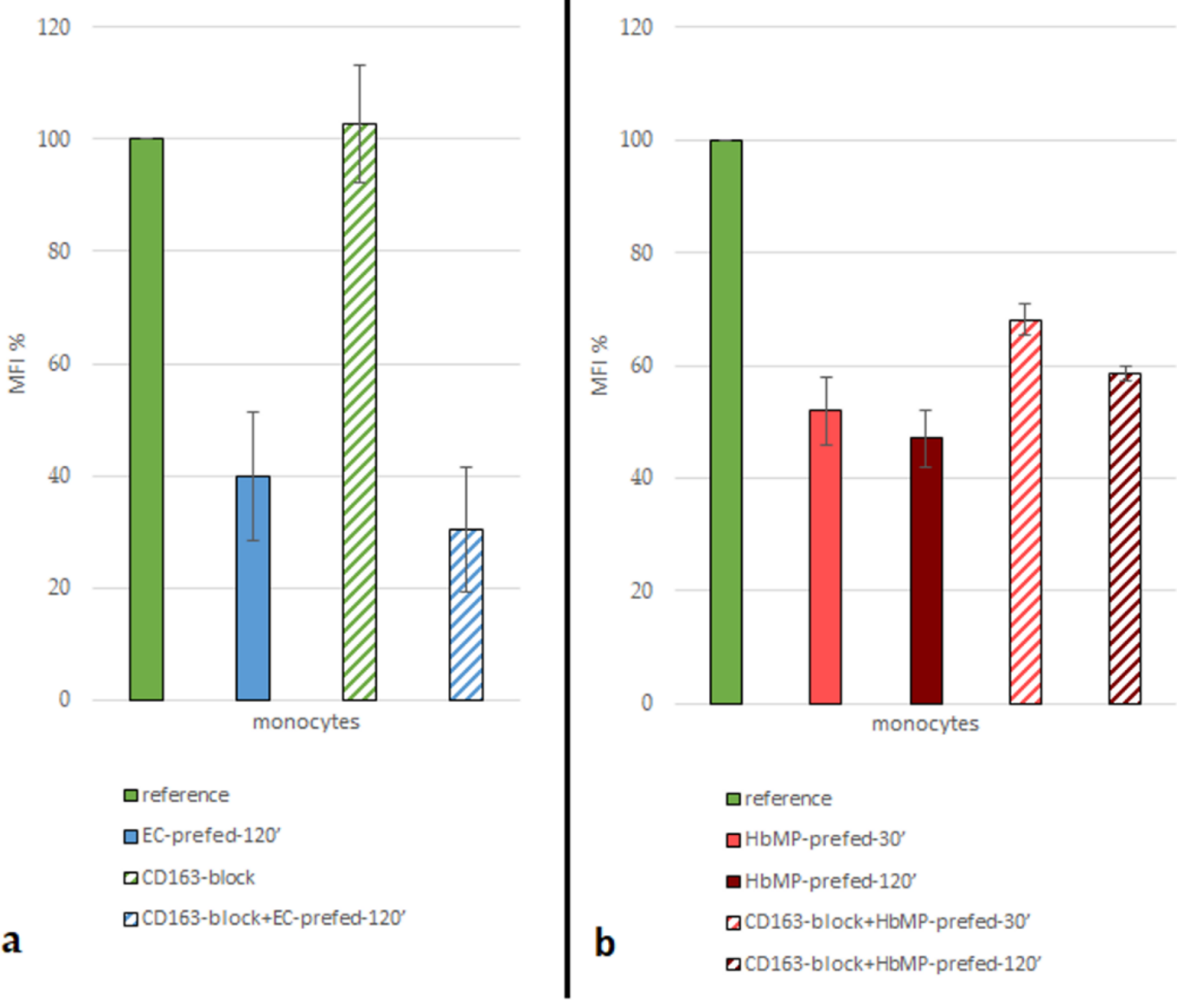
(a) Reduction of normalized MFI (%) by pre-feeding cells with unlabeled *E. coli*; antibody blockade of CD163 does not obstruct the uptake of untagged *E. coli*; *N* = 3. (b) Monocyte-MFI (normalized) after uptake of FITC-tagged *E. coli*; reduced MFI (normalized) after pre-feeding with HbMP. Antibody blockade of CD163 does obstruct the uptake of HbMP; *N* = 3.

Monocyte functionality was confirmed with a reference sample, and the maximum MFI was established. Phagocytes from whole blood were able to take up FITC-labeled *E. coli* lysate unhindered for 10 min at 37 °C (reference). The monocyte population showed a distinct, intact phagocytosis ability (79.3% ± 9.5% FITC-positive monocytes). Keeping the cells at a temperature of 0 °C as negative controls, effectively prevented phagocytic activity (average MFI: 42.3 ± 7.4; reduction in respect to reference: 90.6% ± 1.6%). If the cells were allowed to phagocytose unlabeled *E. coli* lysate for a period of 2 h beforehand and only thereafter were allowed to ingest FITC-labeled *E. coli* lysate, the MFI was reduced to 39.8% ± 19.8% (EC-prefed-120′, Figure 2a). This circumstance indirectly represents phagocytic activity by monocytes: the lower the MFI, the greater the extent of phagocytosis in the “pre-feeding-step”. Covering the monocytes membrane protein CD163 with a specific anti-CD163 antibody had no inhibitory effect on phagocytosis of FITC-labeled (AB-block) and unlabeled *E. coli* lysate (AB-block-EC-prefed-120′).

Pre-feeding the cells with our HbMPs for 120 min (HbMP-prefed-120′) resulted in a reduction of MFI (47.1% ± 8.8%), slightly lower than the MFI reduction that occurred after pre-feeding with unlabeled *E. coli* lysate (39.8% ± 19.8%, EC-prefed-120′). When cells were incubated with HbMPs for a period of 30 min (HbMP-prefed-30′), there was a signal reduction to 51.9% ± 10.3% of the reference value (Figure 2b).

Blockade of CD163 by specific antibodies (AB-blocked-HbMP-prefed-30′; AB-blocked-HbMP-prefed-120′) resulted in an increase of MFI due to reduced HbMP uptake by monocytes. When antibody-covered cells were incubated with HbMPs for 30 min, MFI relative to reference increased by 31.3% compared to unhindered HbMP pre-feeding. When incubated for 120 min, MFI relative to reference increased by 24.2% compared to unhindered HbMP pre-feeding (HbMP-prefed-30′; HbMP-prefed-120′).

### Indirect phagocytosis test no. 2

**Figure 3:**
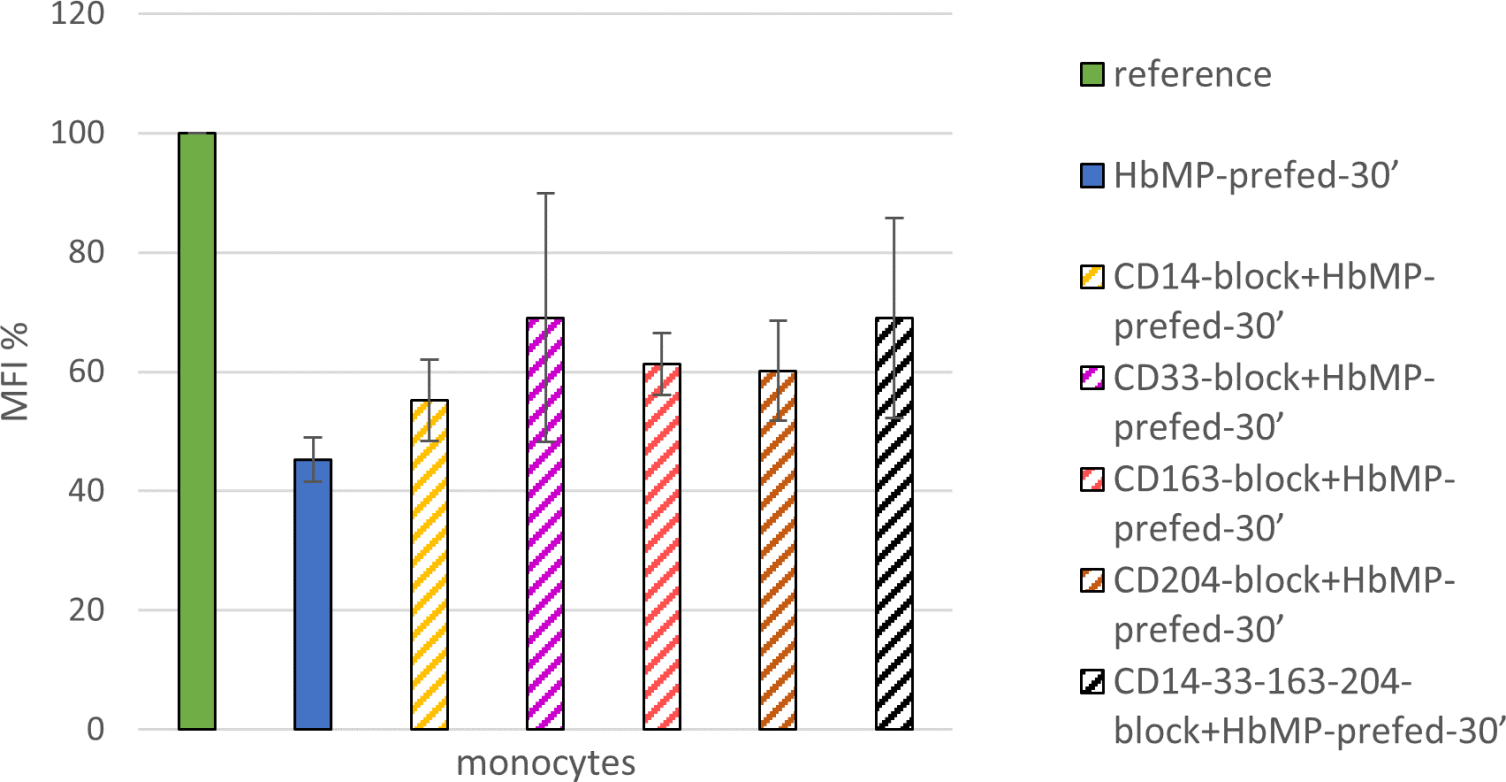
Effect of blockade of CD14, CD33, CD163, and CD204 on HbMP uptake by monocytes; *N* = 3.

In the same manner as described above, a reference sample was prepared to determine the respective maximum FITC value. 85.1% ± 8.9% FITC-positive monocytes indicated an intact monocytic phagocytosis capacity. Pre-feeding the cells with HbMPs limited the uptake of FITC-labeled *E. coli* bacteria and resulted in an MFI reduction to 45.2% ± 3.7% of the reference value. In further samples, the monocytic surface antigens CD14, CD33, CD163, and CD204, or all of them, were blocked by specific antibodies (CD14-block+HbMP-prefed-30′, CD33-block+HbMP-prefed-30′, CD163-block+HbMP-prefed-30′, CD204-block+HbMP-prefed-30′, and CD14-33-163-204-block+HbMP-prefed-30′). After an incubation period of 30 min protected from light and at room temperature, incubation with HbMPs was performed for a period of 30 min at 37 °C, followed by incubation with FITC-labeled *E. coli* for a period of 10 min at 37 °C. The phagocytosis phases were each interrupted by rapid cooling on ice. Leaving the samples at 0 °C throughout the process resulted in an average MFI reduction to 9.4% ± 1.6% of the reference value (MFI: 42.3 ± 7.4). Blocking the monocyte surface antigens resulted in MFI reduction to values between 55.2% ± 6.9% (CD14-block+HbMP-prefed-30′) and 69.0% ± 20.9% (CD33-block+HbMP-prefed-30′) of the reference value. Thus, the blockade of each of the tested receptors showed an inhibitory effect on the uptake of HbMPs compared to unopposed HbMP uptake (HbMP-prefed-30′), as illustrated in Figure 3.

**Figure 4:**
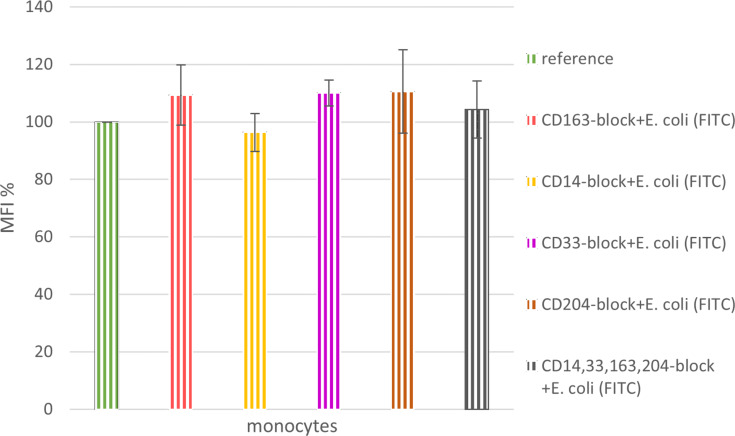
Non-interference of utilized antibodies with the uptake of FITC-tagged *E. coli*; *N* = 3.

As a measure to rule out confounders, we tested for the non-interference of the antibodies on the ability of monocytes to take up FITC-labeled *E. coli* bacteria. In this manner we could confirm the applicability of the chosen test method. Samples with an addition of anti-CD14, anti-CD33, anti-CD163, anti-CD204, or all of the above were prepared as described before. Skipping the HbMP pre-feeding step, the cells were allowed to take up FITC-tagged *E. coli* for 10 min at 37 °C right away. An MFI value in flow cytometry close to the one of the reference sample showed that neither one of the antibodies we used had an effect on the uptake of FITC-labeled *E. coli* bacteria (Figure 4).

## Discussion

Indirect phagocytosis assays were carried out with whole blood in the study presented here. Thus, monocytes are less stressed, and the entire test system can better simulate the in vivo situation. For the negative control samples, it can be assumed that keeping the samples on ice effectively reduced the cell metabolism, and no phagocytosis occurred in these samples (Supporting Information File 1, Figure S2). As expected, the reference samples (reference and CD163-block) showed by far the highest MFI reading. This allowed the remaining values to be considered in their relation to this positive control. Blockade of any of the tested monocytic surface antigens by antibody binding (CD14-block, CD33-block, CD163-block, CD204-block, all antibodies simultaneously CD14,33,163,204-block), had no inhibitory effect on the ability of monocytes to phagocytose FITC-labeled *E. coli* (Figure 4). This observation confirmed the soundness of the indirect measurement method chosen to study the phagocytosis of HbMPs and the role of CD163 in comparison with the other tested receptors. When monocytes with blocked CD163 were pre-fed with untagged *E. coli* lysate (CD163-block+EC-prefed-120′), they took up insignificantly less FITC-labeled *E. coli* lysate compared with non-CD163-blocked monocytes. It can be concluded from the data, that the antibody blockade of CD163 did not obstruct the phagocytosis of untagged *E. coli* in the pre-feeding step. Pre-feeding of monocytes with HbMPs for an incubation time of only 30 min (HbMP-prefed-30′) already caused a reduction of MFI to 50.7% ± 5.1% (mean over indirect phagocytosis tests no. 1 and no. 2) compared to the reference sample. A longer incubation period of 120 min (HbMP-prefed-120′) resulted in an MFI of 47.1% ± 5.08% (indirect phagocytosis test no. 1) of the reference value. This suggests that the Hb particles were taken up with great affinity by the monocytes, a large proportion already within the first 30 min of incubation of cells with HbMPs.

**Figure 5:**
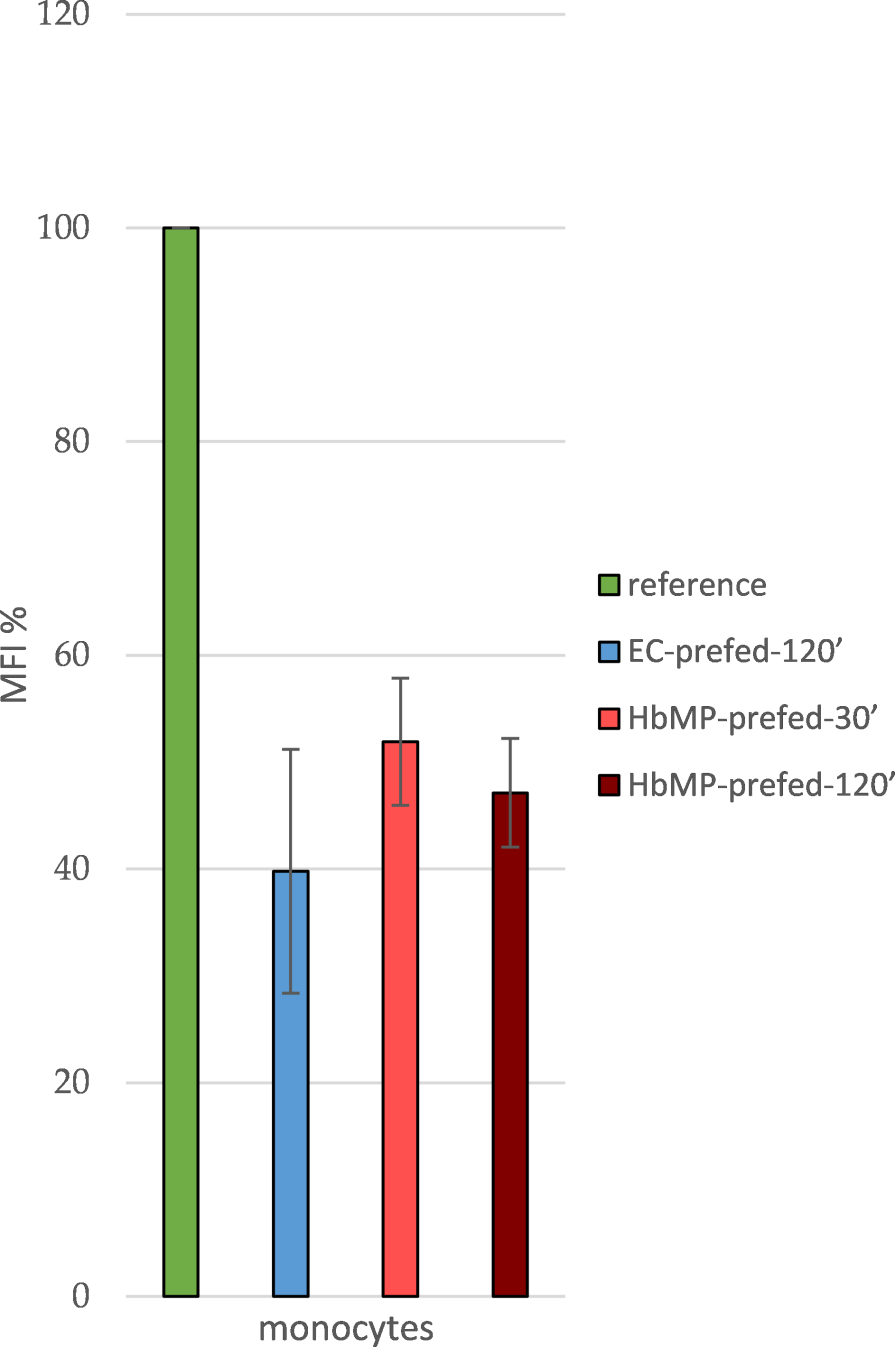
Different uptake of untagged *E. coli* (39.8 ± 11.4%) vs HbMP (30′: 51.9 ± 6.0%; 120′: 47.1 ± 5.1%) by monocytes; *N* = 3.

**Figure 6:**
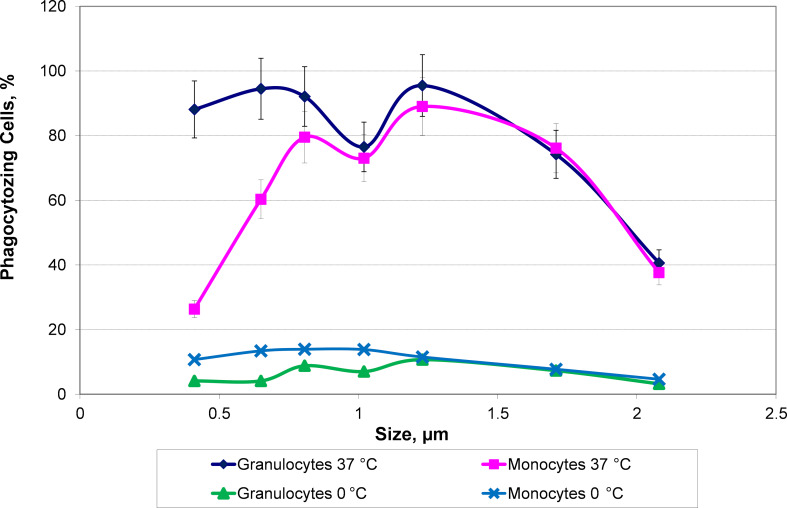
Phagocytosis of PMMA-FluoroGreen-COOH-MP diameter between 0.4 and 2.1 µm by monocytes and granulocytes. *N* = 3.

**Figure 7:**
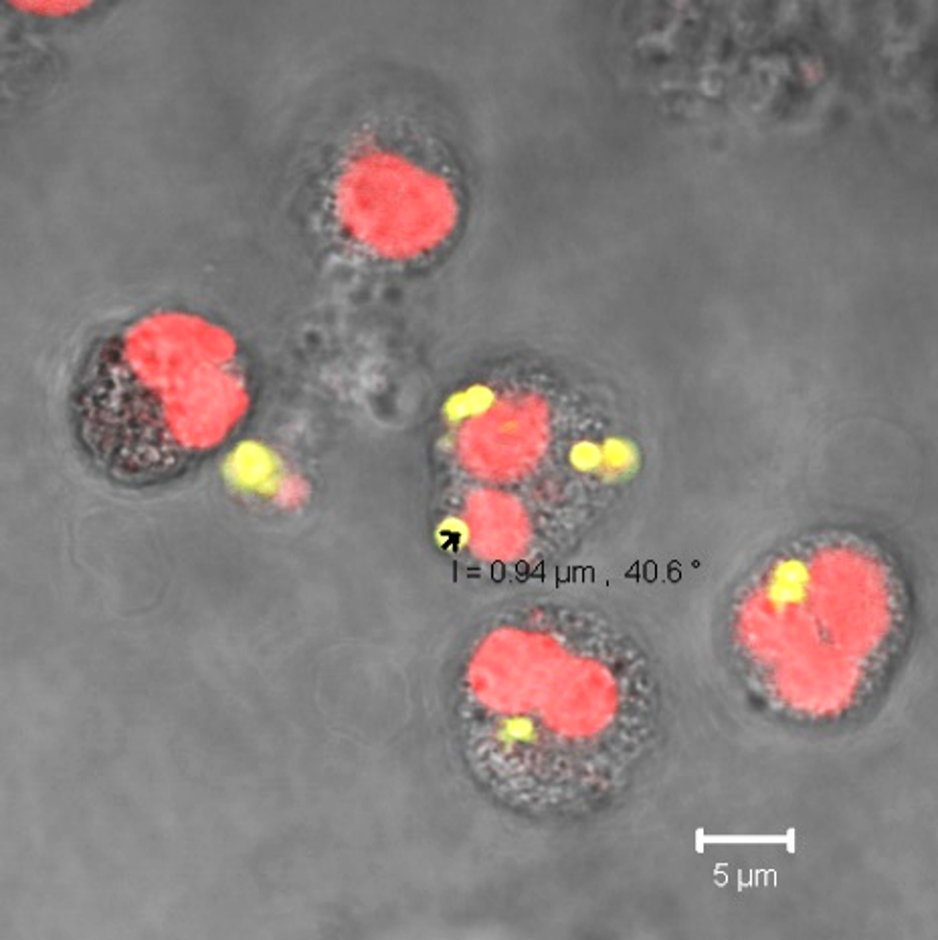
The confocal laser scanning microscopy image showing the uptake of FITC-labeled MPs (green) after incubation at 37 °C for 30 min. The DNA of leukocytes was stained with propidium iodide (red).

Blockade of CD163 prior to the incubation of the cells with HbMPs resulted in an increased MFI after feeding with FITC-labeled *E. coli* (CD163-block+HbMP-prefed-120′ vs HbMP-prefed-120′, Figure 2; Table 4). It can be concluded, that less HbMPs have been taken up by monocytes in pre-feeding due to the blockade of CD163, with a maximum reduction of uptake of 31.3% ± 10.4%. Thus, CD163 does appear to play an important role in the phagocytosis of our HbMPs by monocytes. These results are also in line with previous research. In several other studies, using various other HBOCs, a direct Hb–CD163 interaction was observed [5,27,39]. However, the blockade of CD163 could not completely inhibit the uptake of HbMPs, which suggests the existence of at least one further mechanism for HbMP phagocytosis. Screening for possible other players in the monocytic uptake of HbMPs, we observed that CD14, CD33, and CD204 all showed an effect on phagocytic activity when blocked by antibodies, which ranged from 10% (CD14-block+HbMP-prfed-30′) to 24% (CD33-block+HbMP-prefed-30′) lower HbMP uptake, compared to unhindered HbMP uptake (HbMP-prefed-30′, Figure 3). However, the observed effects do not seem to cumulate when the tested antigens were blocked all at once (CD14,33,163,204-block+HbMP-prefed-30′). Overall, while each of the receptors we tested seems to contribute in some way to the phagocytosis of HbMPs, neither of them alone provides a satisfactory explanation for the full extent of this process. The overall extent of phagocytosis of HbMPs appears to be slightly lower to that of untagged *E. coli* lysate, as Figure 5 shows. It is conceivable, that the reason for the uptake of HbMPs by monocytes might not be their Hb content. In their work from 2013, Yan et al. [40] showed how the formation of a protein corona influences particle–cell interactions. Especially bovine serum albumin (BSA) showed an ambivalent effect. On the one hand, the corona, which consisted mainly of BSA, reduced the direct cell surface adhesion of the test particles. On the other hand, binding to the particles caused a conformational change within the structure of BSA. This denatured BSA promoted binding by scavenger receptor A (SR-A/CD204) and, thus, induced internalization of the protein–particle–receptor complex. In the case of our HbMPs containing human serum albumin, we assume that a conformational change within HSA might occur when the molecules are cross-linked by glutaraldehyde. Detection of this denatured protein by receptors of phagocytic cells would also be conceivable in this case to explain CD204’s part in the uptake of HbMPs. Further research on this topic will be conducted in the future. To be considered as well is a possible mechanism leading to particle uptake by phagocytes based on the particle size. Our own results regarding PMMA-FluoroGreen-COOH particles (microparticles GmbH, Berlin, Germany) with a diameter between 0.4 and 2.1 µm demonstrate this relationship, as Figure 6 and Figure 7 illustrate (incubation of heparin blood with 2 × 10^8^ MP/mL) [41].

**Table 4:** Overview of phagotest no. 2 samples, normalized MFI values.

sample^a^	normalized MFI ± SEM, %

reference	100.0 ± 0
HbMP-prefed-30′	45.2 ± 3.7
CD14-block-HbMP-prefed-30′	55.2 ± 6.9
CD33-block-HbMP-prefed-30′	69.0 ± 20.9
CD163-block-HbMP-prefed-30′	61.2 ± 5.2
CD204-block-HbMP-prefed-30′	60.2 ± 8.3
CD14,33,163,204-block-HbMP-prefed-30′	68.9 ± 16.8

^a^Blocking monocytic CD14, CD33, CD163, and CD204 reduces HbMP uptake by monocytes (MFI is inversely proportional to the extent of uptake in pre-feeding step); *N* = 3.

Champion et al. researched this relationship in a model as well [42]. Test particles (polystyrene microspheres) with diameters in the range of 3 µm were phagocytosed to a higher extent than both smaller and larger particles in this experiment. According to the authors, this is based on the number of possible contact points with the cells, depending on the morphology of the cell surface. In addition, the particle charge (i.e., hydrophilicity/hydrophobicity) also played a role. Together with the particle composition, this might be a possible explanation for the difference in the results of Champion et al. and our own. Buehler et al. observed a dependence on particle size in their studies of direct Hb/HBOC–CD163 interaction. HBOCs from both bovine and human Hb showed similar Hp-independent uptake via CD163 when prepared in a similar manner (α chain-polymerized bovine Hb and β chain-polymerized HbA0). After fractionation of the HBOCs and repetition of the experiment, a clear size dependence of the affinity of CD163 for individual HBOC fractions (32 kDa to >600 kDa) was shown. Also polymerization, cross-linking, and cross-linking sites influenced the receptor’s affinity for individual HBOCs, while the origin of the utilized Hb seemed to be of no relevance [16]. Furthermore size dependence probably exists also in antibody-induced phagocytosis, as Montel et al. were able to show [43]. However, it remains unclear whether the various mechanisms that may be at play follow a hierarchy. It is also unclear which processes might be responsible for the overall extent of phagocytosis, over which period of time they take place, and whether processes take place in parallel. Other receptors and ways of particle recognition might affect the affinity with which monocytes take up HbMPs. Another mechanism that has received little attention in research to date is the opsonization of particles by complement factors. Especially factor C3b, which can arise spontaneously from C3 by hydrolysis, could be considered here. Moghimi et al. report numerous possible interactions of the complement system with, among others, nanoparticles as a drug delivery system [44]. The authors also discussed the possible effects of spontaneously forming water shells and emerging hydrogen bonds, altered surface structures, and possible interactions between proteins and particles, in particular polymers such as HbMPs [45–46]. The adsorption of various plasma proteins seems to not only enhance the chance of complement activation but also to promote phagocytosis directly, as Zhang describes [47]. Lück et al. showed by two-dimensional electrophoresis that upon serum incubation, protein accumulation occurred on latex particles with an average size of 660 nm leading to complement activation [48]. At this time, it is unclear whether this mechanism could also play a role in HbMP sequestration as the HSA on the surface of our particles has shown to attenuate the adsorption of plasma proteins [19].

Kloypan et al. observed granulocytic uptake of albumin-based particles in an indirect phagocytosis assay [22]. An observation that was made repeatedly in the present study. Thus, it appears that Hb is not necessarily the key factor that triggers uptake into cells and, in any case, CD163 does not appear to be the single key protein for this either. Xiong et al. observed agglomeration of HBOCs in the liver of a rat model after intravenous injection of a HBOC solution, which became visible after a short while in the MRI scan [24]. While the authors hypothesized that the HBOC was taken up by CD163-expressing Kupffer cells/macrophages, Chow et al. reported that when isolated rat livers were perfused with a HBOC solution, hepatocytes also took up abundant hemin, as determined by heme oxygenase-1 expression [25]. Goldfischer et al. observed immunohistochemically the presence of Hb in phagocytic but also hepatocytic lysosomes [49]. Hepatocytes, however, do not express CD163; therefore; they must have a currently still unknown mechanism potentially for Hb recognition, but surely for HBOC uptake. Future research will have to show whether this mechanism might also be responsible for a part of the uptake of HBOCs in monocytes and macrophages.

## Conclusion

With this study, the role of CD163 in the phagocytosis of hemins by monocytes could be confirmed also for our HbMPs, namely GA-polymerized bovine Hb sub-micron particles coated in HSA. Moreover, our data suggest the involvement of other monocytic surface proteins in this process as well as a possible size influence on particle uptake.

## Supporting Information

File 1 Supplementary data.

## Data Availability Statement

Data are available after contacting the corresponding author and stored on the central server of Charité - Universitätsmedizin Berlin.
