# Supplementary material for: Recognition mechanisms of hemoglobin particles by monocytes – CD163 may just be one
Source: Beilstein J Nanotechnol. 2023 Oct 19;14:1028–40. doi: 10.3762/bjnano.14.85 (PMC10616704; doi:10.3762/bjnano.14.85)
Supplement: File 1 — Supplementary data. [file Beilstein_J_Nanotechnol-14-1028-s001.pdf]

BEILSTEIN JOURNAL OF NANOTECHNOLOGY

Supporting Information

for

Recognition mechanisms of hemoglobin particles by monocytes – CD163 may just be one

Jonathan-Gabriel Nimz, Pichayut Rerkshanandana, Chiraphat Kloypan, Ulrich Kalus, Saranya Chaiwaree, Axel Pruß, Radostina Georgieva, Yu Xiong and Hans Bäumler

Beilstein J. Nanotechnol. **2023,** *14,* 1028–1040. doi:10.3762/bjnano.14.85

Supplementary data

License and Terms: This is a supporting information file under the terms of the Creative Commons Attribution License (https://creativecommons.org/licenses/by/4.0). Please note that the reuse, redistribution and reproduction in particular requires that the author(s) and source are credited and that individual graphics may be subject to special legal provisions.
The license is subject to the *Beilstein Journal of Nanotechnology* terms and conditions: (https://www.beilstein-journals.org/bjnano/terms)

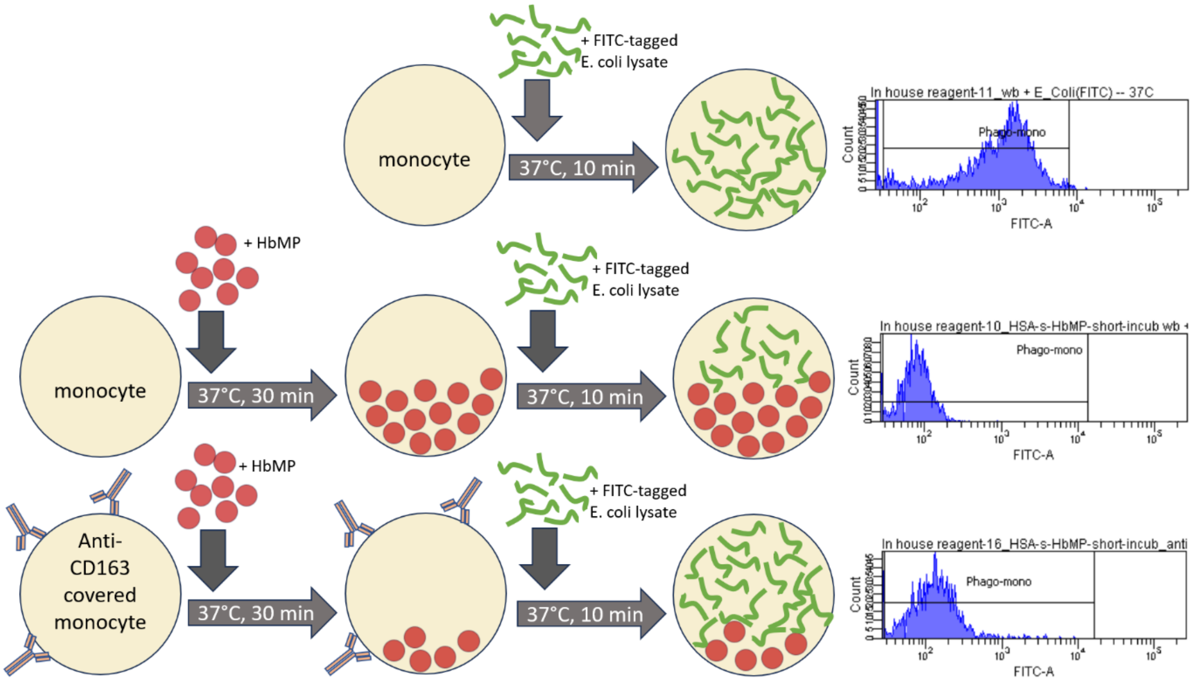

Figure S1: Test principle, MFI depending on the uptake of FITC labeled *E. coli* lysate. Uptake of FITC labelled *E. coli* lysate depending on the extent of phagocytosis in pre-feeding step.

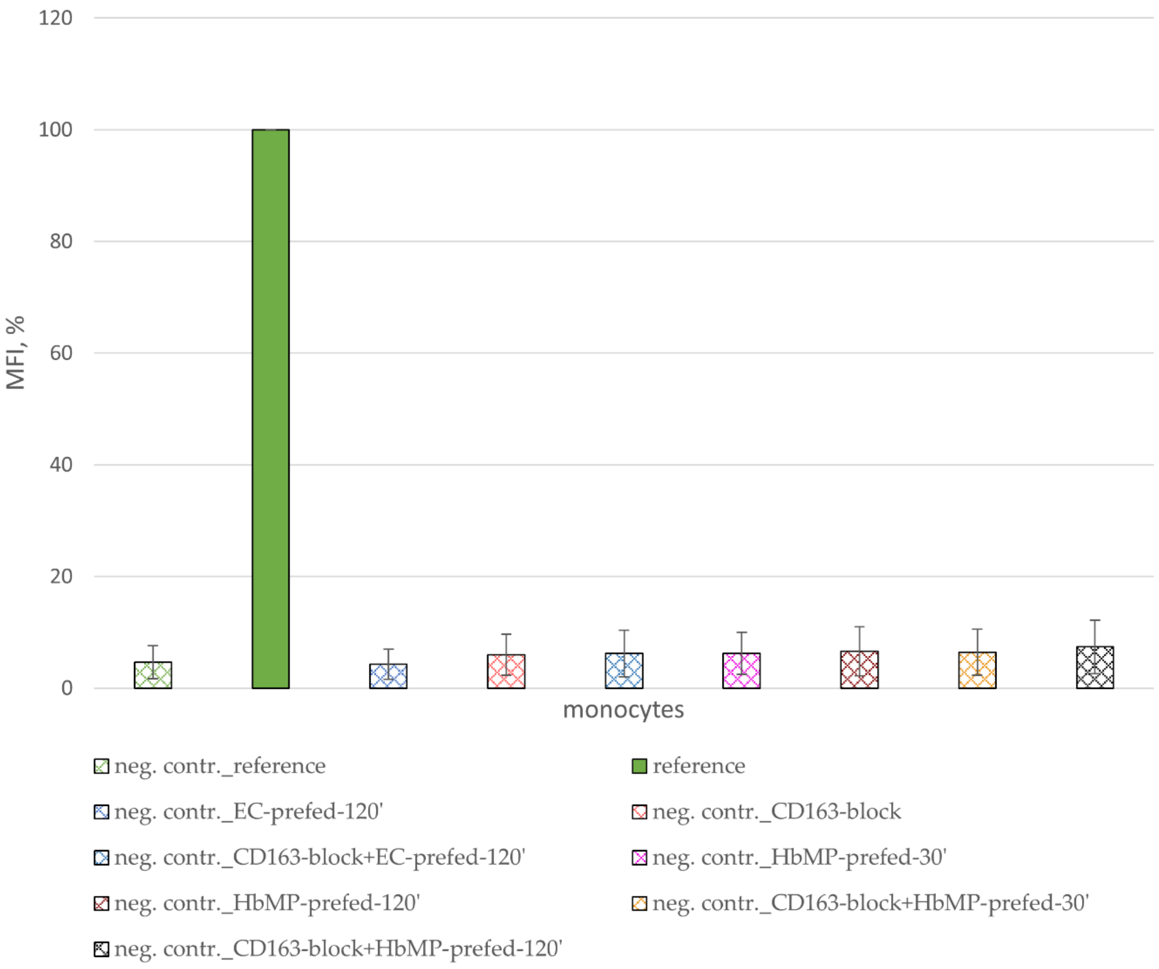

Figure S2: negative control-samples, normalized MFI, monocytes; *N* = 3.

Table S1: Overview of phagocytosis-test No1 samples, normalized MFI-values, monocytes.

**sample**	**normalized MFI ± SEM, %**
reference	100.0 ± 0
EC-prefed-120’	39.8 ± 11.4
CD163-block	102.6 ± 10.5
CD163-block-EC-prefed-120’	30.3 ± 11.1
HbMP-prefed-30’	51.9 ± 6.0
HbMP-prefed-120’	47.1 ± 5.1
CD163-block-HbMP-prefed-30’	68.1 ± 2.9
CD163-block-HbMP-prefed-120’	58.5 ± 2.1

blocking monocytic CD163, reduces HbMP-uptake by monocytes (MFI is inversely proportional to the extent of uptake in pre-feeding step); $N = 3$.
